# Treatment Options for Unilateral Agenesis of the Maxillary Lateral Incisor Combined with Contralateral Microdontic or Peg-Shaped Lateral Incisor: A Systematic Review

**DOI:** 10.3390/dj13040169

**Published:** 2025-04-17

**Authors:** Federica Bitonto, Alessio Verdecchia, Massimiliano Lombardo, Erica Lipani, Claudia Dettori, Enrico Spinas

**Affiliations:** 1 Department of Surgical Sciences, Postgraduate School in Orthodontics, University of Cagliari, 09124 Cagliari, Italy; federibit@gmail.com (F.B.); info@massimilianolombardo.com (M.L.); erica.lipani@outlook.it (E.L.); 2 Department of Surgical Sciences, School of Dental Medicine, University of Cagliari, 09124 Cagliari, Italy; claudia.dettori@gmail.com

**Keywords:** lateral incisor agenesis, maxillary lateral incisor, peg-shaped lateral incisor, microdontia, orthodontic treatment

## Abstract

**Background:** Managing unilateral maxillary lateral incisor agenesis (UMLIA) with an associated microdontic or peg-shaped contralateral incisor (Mi or Peg MLI) presents clinical and esthetic challenges. Deciding between orthodontic space opening (OSO) or closure (OSC) and whether to restore or extract the Mi or Peg MLI are critical factors for achieving optimal treatment outcomes. **Methods:** A systematic review was conducted using a dual strategy across Scopus, Web of Science, Embase, PubMed, and Cochrane Central Register of Controlled Trials databases. The Cochrane RoB2 tool was used for randomized controlled trials (RCTs), the ACROBAT-NRSI tool for non-randomized studies, and the JBI Manual for case reports. High-quality case reports were included when the literature was limited. The GRADE (Grading of Recommendations, Assessment, Development, and Evaluation) tool evaluated the certainty of evidence, considering bias, inconsistency, indirectness, imprecision and other considerations. **Results:** Thirty-five studies met the inclusion criteria, assessing treatment modalities, esthetic outcomes, periodontal health, and occlusal and temporomandibular joint (TMJ) function. Both OSO and OSC demonstrated similar occlusal and TMJ outcomes, while slight differences in esthetic and periodontal results were noted between the two approaches. Direct composite and indirect ceramic restorations showed comparable effectiveness for reshaping the Mi or Peg MLI. Extraction combined with OSC contributed to improved smile symmetry. **Conclusions:** A personalized, multidisciplinary approach is essential for treating UMLIA with an Mi or Peg MLI. Both OSO and OSC are viable options, and the choice should be tailored to the patient’s specific clinical scenario, with attention to achieving the best esthetic and periodontal outcomes. Further research is needed to refine treatment protocols and support clinical decision-making.

## 1. Introduction

The congenital absence of an upper lateral incisor, known as unilateral maxillary lateral incisor agenesis (UMLIA), represents a significant clinical challenge in orthodontics, particularly when it is associated with a microdontic or peg-shaped contralateral maxillary lateral incisor (Mi or Peg MLI). Approximately 20% of all dental agenesis in Caucasian population cases involve the upper lateral incisors (MLIs), making this condition the second most common type after mandibular second premolars, excluding third molars [1]. According to Polder’s research [2], women are more likely to experience this condition than men, with an odds ratio of 1.22 (95% CI: 1.14 to 1.30) and bilateral agenesis of the maxillary lateral incisors (BMLIA) is more prevalent (95% CI = 50.9–57.0%) compared to UMLIA.

The etiology of lateral incisor agenesis is multifactorial, involving genetic, epigenetic, and environmental factors. Genes such as MSX1 and PAX9 have been implicated in the development of this condition, and their mutations are often associated with both the agenesis of teeth and anomalies in tooth size and shape [3]. This is the reason why, in patients with UMLIA, several studies have observed a higher prevalence of Mi or Peg MLI, with percentages ranging from approximately 20.2% [4,5] to 38.8% [6] and up to 44.9% [7]. The association of those two conditions affects both the physical and psychological well-being of patients [8]. Current research [9,10,11,12,13,14] suggests two primary strategies for UMLIA: orthodontic space closure (OSC) with or without composite tooth re-contouring (TR) of the canine and premolar, or orthodontic space opening (OSO) followed by prosthetic replacement with dental implants (IT), fixed partial dentures (FDPs) or resin bonded bridges (RBBs). Simultaneously, the Mi or Peg MLI can be managed through restoration, composite resin (CR) or ceramic veneer (CV) or extraction with OSC. However, a lack of high-quality comparative studies makes it difficult to establish clear guidelines for treatment selection.

Hypothesis and study rationale: This study hypothesizes that both OSC and OSO, when combined with appropriate management of the Mi or Peg MLI, can provide favorable functional and esthetic outcomes. The rationale behind this systematic review is to evaluate and compare available treatment modalities to assist clinicians in optimizing treatment planning.

## 2. Materials and Methods

### 2.1. Protocol and Registration

The present systematic review was conducted following the guidelines for Systematic Reviews and Meta-analyses (PRISMA) [15].

The protocol was registered in the PROSPERO database under the number CRD42024622872.

### 2.2. Information Sources and Search Strategy

A two-part search strategy was implemented on 1 December 2024 across five databases—Scopus, Web of Science, Embase, PubMed, and the Cochrane Central Register of Controlled Trials—to ensure comprehensive analysis. The first search focused exclusively on orthodontic and prosthetic options for UMLIA, while the second examined treatments for Mi or Peg. This division was necessary due to the limited literature covering both conditions simultaneously.

Additionally, research was conducted in gray literature using the OpenGrey database to ensure comprehensive coverage of relevant studies. The complete search strategy for all databases is provided in Tables S1 and S2 in the Supplementary Materials.

### 2.3. Study Selection and Eligibility Criteria

To guide the research strategy, the following PICO question was formulated:

- **P (Population)**: patients with UMLIA and a contralateral Mi or Peg MLI.
- **I (Intervention)**: orthodontic and prosthodontic treatments.
- **C (Comparison)**: different treatment options:
  1. Bilateral orthodontic space closure with extraction of the contralateral Mi or Peg MLI;
  2. Unilateral orthodontic space closure with or without composite tooth re-contouring of the canine and premolar on the side of UMLIA, combined with restoration of the contralateral Mi or Peg MLI using direct composite or ceramic veneer;
  3. Unilateral orthodontic space opening with implant treatment or fixed dental prosthesis on the side of UMLIA with restoration of the contralateral Mi or Peg MLI using direct composite or ceramic veneer.
- **O (Outcome)**: identification of the most recommended treatment approach based on primary outcomes including esthetics, periodontal status, occlusion and temporomandibular joint function.

The inclusion criteria were as follows:

- Studies on UMLIA (with or without Mi or Peg MLI).
- Studies on Mi or Peg (with or without UMLIA).
- Studies evaluating orthodontic and/or prosthetic treatment modalities
- Randomized controlled trials, cohort studies, and case–control studies.
- High-quality case reports (only for the second research phase, due to the lack of other higher-level evidence).

In the second research phase, the inclusion of case reports was a deliberate methodological choice to address the scarcity of studies with a higher level of scientific evidence. These case reports were carefully selected using an adapted qualitative scale [16] ensuring only the most relevant and well-structured studies were included.

The exclusion criteria were as follows:

- Studies involving BMLIA.
- Studies involving syndromes, cleft lip/palate cases.
- Studies investigating trauma-induced or caries-related lateral incisor absence.
- Animal or in vitro studies.
- Review studies.

No restrictions were applied regarding the publication year or language.

### 2.4. Selection Process

Duplicates were removed using Zotero (version 6.0.36) and then manually verified. Two different reviewers (FB and AV) independently assessed and selected relevant studies based on their titles and abstracts. To assess the level of agreement among the reviewers, Cohen’s kappa coefficient (K: 0.66) [17] was calculated, indicating substantial agreement. In case of disagreement, a third reviewer was consulted (ML). Authors showed a substantial agreement (Cohen’s kappa: 0.66). After reviewing the full texts, studies were chosen for inclusion in the review according to the established inclusion and exclusion criteria.

### 2.5. Data Collection

The following data were extracted from the selected studies:

- First author and year of publication.
- Country of origin.
- Study design.
- Sample characteristics (sex and age).
- Dental analysis (Bolton or dento-basal discrepancy).
- Type of agenesis (UMLIA/BMILIA).
- Type of anomaly affecting the lateral incisor (Mi or Peg).
- Diagnostic tests used (photography, radiographies, diagnostic casts, diagnostic wax-up, intraoral mock-up).

The primary outcomes were esthetics, periodontal status and occlusion, and temporomandibular joint function following treatment, while the secondary outcome was the follow-up period.

Esthetic assessment in the first search focused on ten specific criteria: smile arc, ratio and symmetry of the maxillary central incisors, proportion between the anterior upper teeth, presence of anterior upper spaces, gingival design, gingival exposure, buccal corridors, tooth color and anatomical shape, and lip volume [18]. In the second search, the esthetic evaluation of the treatment, based on the type of material used for Mi or Peg MLI restoration, included color match, marginal discoloration and adaptation, surface roughness and integrity [19].

Periodontal status was evaluated through indicators such as bleeding on probing, gingival recession, and papillae formation, while TMJ status considered symptoms like headache, joint noises, and unilateral chewing.

### 2.6. Quality Assessment

The quality assessment was carried out by two independent reviewers (FB and EL), who evaluated the included studies separately, with disagreements resolved by consensus or by a third reviewer (ES). Due to the heterogeneity of the included studies, different tools were employed for risk-of-bias assessment.

The risk of bias in RCTs was evaluated using the Cochrane Collaboration’s RoB 2 tool [20] across five domains: randomization process, deviation from intended interventions, missing outcome data, measurement of the outcome and selection of the reported result. Based on the assessment, RCTs were categorized as having low risk, some concerns or high risk.

For prospective and retrospective studies, the Cochrane Risk Of Bias Assessment Tool for Non-Randomized Studies of Interventions (ACROBAT-NRSI) was used [21]. Seven domains were considered: bias due to confounding, bias in selection of participants into the study, bias in measurement of interventions, bias due to departures from intended interventions, bias due to missing data, bias in measurement of outcomes, and bias in selection of the reported result. Based on the information provided for each study, the risk of bias was classified as low, moderate, serious, critical or NI (no information on which to base a judgment about risk of bias).

For the second stage, a quality assessment tool based on the JBI Manual for Evidence Synthesis [16] was used to evaluate the quality and reliability of the content of case reports assessed for eligibility, allowing for the inclusion of content with greater relevance and better structure. Eight structured questions assessed the completeness of patient data, diagnostic tests, post-intervention condition, adverse events and key takeaways. Studies were scored based on ‘Yes’ responses, with a score of 5 or higher indicating high quality, and with a score below 5 indicating low quality. The GRADE tool for formulating and grading recommendations in clinical practice was also employed [22].

## 3. Results

### 3.1. Literature Search and Screening Process

**Figure 1:**
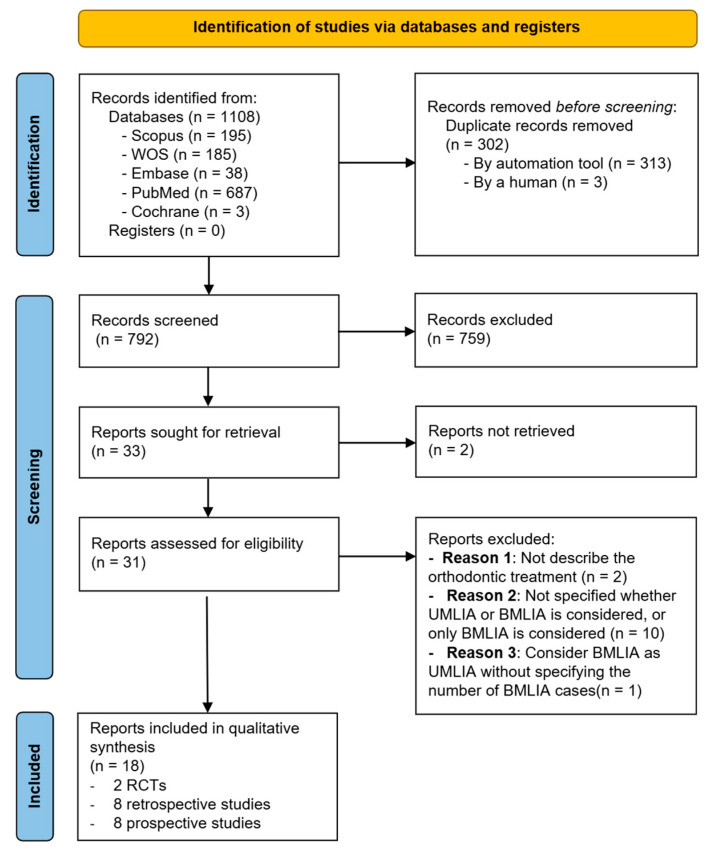
Flow chart showing the literature search and selection for the first research stage.

The first search yielded a total of 1108 results: 195 from Scopus, 185 from Web of Science, 38 from Embase, 687 from PubMed, 3 from Cochrane Central Register of Controlled Trials and 0 from OpenGrey. After removing duplicates, 792 articles remained. Subsequently, after reading the title and abstract, 33 articles were sought for retrieval; 2 reports were not retrieved, so only 31 underwent full-text evaluation. Finally, after a thorough assessment, 18 articles met the inclusion criteria and were included in the review. The details of the literature search and selection procedure are shown in a flow chart in Figure 1.

**Figure 2:**
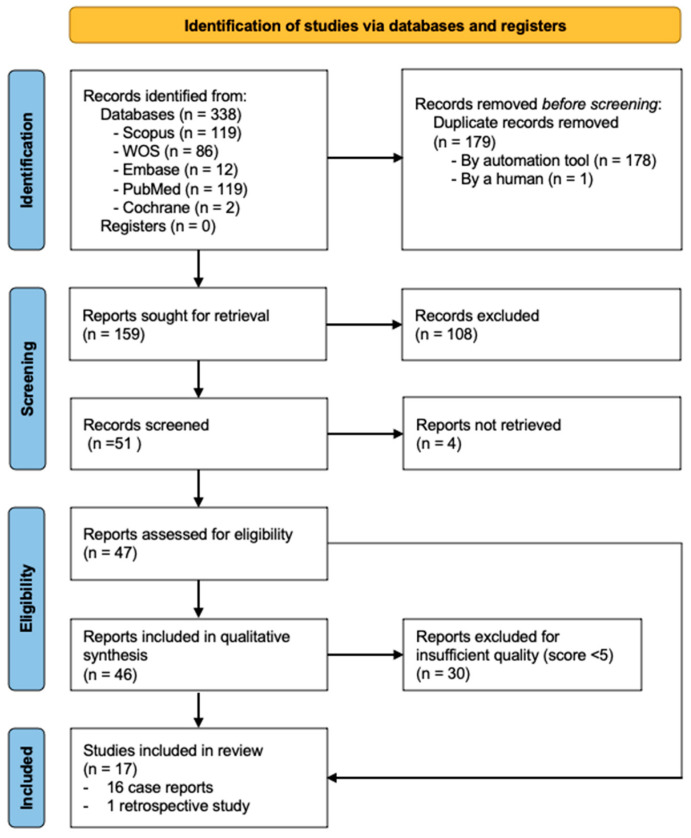
Flow chart showing the literature search and selection for research stage 2.

The second search generated a total of 338 results: 119 from Scopus, 86 from Web of Science, 12 from Embase, 119 from PubMed, 2 from Cochrane Central Register of Controlled Trials and 0 from OpenGrey. After removing duplicates, 159 articles remained. Subsequently, after reading the title and abstract, 51 articles underwent full-text evaluation. Finally, after a thorough assessment, 17 articles met the inclusion criteria and were included in the review (Figure 2).

To conclude, a total of 35 articles were included in this review, combing the results of the two searches.

### 3.2. Description of the Included Studies for Research Stage 1

Table 1 describes the main characteristics of the studies included in the first research stage of this systematic review.

This systematic review includes studies published between 2000 and 2024 from Italy (*n* = 7) [25,30,33,34,35,36,39], Sweden (*n* = 3) [1,27,28], Brazil (*n* = 2) [24,31], the USA (*n* = 2) [23,38], Greece (*n* = 1) [29], the UK (*n* = 1) [26], Denmark (*n* = 1) [32] and Scotland (*n* = 1) [37].

Study design varies, including prospective studies (*n* = 7) [24,30,32,34,35,36,39], randomized controlled trials (*n* = 2) [25,31], and a retrospective study [1,23,26,27,28,29,33,37,38].

Sample sizes ranged from 8 to 72 participants, mostly female, aged 12 to 40 years, with a focus on adolescents (12–18 years) and adults (18–35 years).

Only three studies [23,25,32] focused solely on UMLIA, while the remaining fifteen studies differed in their focus [1,24,26,27,28,29,30,31,32,33,34,35,36,37,38,39].

#### Treatment Approaches

Two studies do not implement orthodontic treatment and directly investigate IT [25,39]. OSO is the most frequently discussed in fourteen studies. Among these, nine studies utilize this space for IT [24,27,28,29,30,32,34,35,38], while four studies employ RBBs [1,26,29,37], one study focuses on fixed partial dentures [36], and one study analyzes OSO alone [23]. OSC is evaluated in nine different studies [1,23,24,27,28,29,31,33,37].

### 3.3. Pre-Treatment Parameters

The pre-treatment parameters of the studies included in the first research stage are collected in Table 2.

Malocclusion classification was rarely detailed, with only a few studies providing specific occlusal information. Most participants were in Class I, with limited cases in Classes II and III. Garnett et al. [26] reported incisor occlusal classes, Kafantaris et al. [29] described mostly Class I patients, and Pithon et al. [31] reported primarily skeletal Class I and Angle Class I/II categories, while Ulhaq et al. [37] noted that most patients had an overjet ≥ 2 mm and were mainly Class I or Class II in molar relationships.

Studies used a combination of 2D and 3D methods, including periapical, panoramic or cephalometric radiographs [1,25,29,30,31,32,33,34,39] as well as CBCT and dental CT scans [23,25,34,35,38,39]. Some studies [25,34,35,39] combined 2D and 3D diagnostics, but many did not specify the methods used [24,26,27,28,36,37].

Most studies did not conduct formal Bolton or dento-basal analysis [24,25,26,27,30,33,34,35,36,38,39]. One study [23] reported reduced crown and root widths of the contralateral lateral incisor in UMLIA patients. Some studies focused on the mesio-distal (MD) space without a formal Bolton analysis. Pithon et al. [31] found MD space of 6.35 mm in the OSC group compared to 6.9 mm in the control. Roccuzzo et al. [32] used implant sizes based on MD space, with 2.9 mm implants for 5.9–6.3 mm spaces and 3.3 mm implants for 6.4–7.1 mm spaces. Ulhaq et al. [37] noted inadequate MD space in 87% of the OSC group and 45% of the OSO group. Robertsson et al. [1] reported systematic differences in space analysis, with the OSC group showing a negative dento-basal mandibular discrepancy and a smaller maxillary quadrant discrepancy (+2.7 mm) compared to the OSO group.

### 3.4. Treatment Outcomes

Table 3 describes the primary and secondary outcomes of different treatment methods for MLIA.

Esthetic outcomes varied significantly among treatments, with several studies not reporting specific data [23,24,26,31,33,37,38,39]. Studies that did assess esthetics indicated better outcomes with OSO followed by IT or RBBs. Crown color satisfaction was higher in IT groups, with no unacceptable cases reported [27,28], while OSC showed up to 21% dissatisfaction. RBBs after OSO also resulted in higher satisfaction (81%) compared to OSC (45%) [1]. Implant size influenced crown color perception, with 47.7% of 2.9 mm implants rated excellent versus 26.2% for 3.3 mm implants [32].

Crown shape satisfaction was 81% with OSO versus 45% with OSC [1]. Soft tissue esthetics showed more non-acceptable gingival color in IT groups (60.5% and 73.5%) than OSC (9% and 3%) [27,28], and according to [25], immediate restoration of the implant has better esthetic results for soft tissue.

Lip closure strain and midline deviation showed no significant differences between treatment types [27,28]. Maxillary anterior teeth symmetry was higher with OSC (67%) compared to OSO (50%) [1]. The 3.3 mm implant group also showed good symmetry (61.9%) [32].

Smiling esthetics were generally satisfactory across all treatment groups [28,30,36].

Periodontal health varied by treatment modality, with plaque index (PI) lower in OSC-treated patients compared to those treated with OSO and IT [24], or compared to those treated with OSO and RBBs [1]. No significant differences were found when using implants with narrower diameters [32]. Probing depth (PD) remained under 4 mm in all groups [24,32,33], with lower BOP in OSC-treated patients compared to OSO and IT groups [24,27,33]. Studies on IT reported stable peri-implant soft tissue health [34,35], with minimal peri-implantitis (2%) [32]. Gingival recession (GR) was slightly higher in OSO and IT patients [27,28] but largely absent in those with a thick periodontal biotype [24]. The papilla index (PaI) indicated better gingival health in OSC-treated patients [24,27,28], with improved outcomes using 3.3 mm implants [32]. Bone loss remained stable across treatment types [25,30,32,38,39].

Thirteen studies did not report TMJ data [23,25,26,27,28,30,31,32,34,36,37,38,39]. Studies that did provide information found a minimal correlation between treatment type and TMD symptoms. Most patients showed no TMD signs post-treatment, regardless of whether OSC or OSO was used [24]. TMD-related symptoms like myofascial pain and disk displacement were rare (<1%) [1].

Occlusal outcomes were generally positive across treatments. Functional group guidance was more common in OSC-treated patients [24,33], while canine lateral guidance appeared more in OSO and IT groups [24].

Both OSO with IT and FRC-FPD treatments provided reliable functional outcomes [34,35,36,39] with no signs of infraocclusion [30].

Studies comparing OSO with IT and OSC [1,27,28] showed no significant differences in occlusal parameters (overjet, overbite, angle classification) except for increased proclination of maxillary incisors with OSO and IT.

Only three studies did not report follow-up [23,37,38], and in Kafantaris et al. [29], only the OSC group lacked follow-up data. Follow-up durations ranged from 1 to 10 years, with short-term studies at 1 year [31,32], medium-term studies at 3–5 years [24,25,27,28,36,39], and long-term studies up to 10 years [1,26,30,33].

### 3.5. Description of the Included Studies for Research Stage 2

Table 4 describes the main characteristics of the reviewed studies included in the second research phase. The included studies were conducted between 2008 and 2024 [40,41,42,43,44,45,46,47,48,49,50,51,52,53,54,55,56]. The reviewed studies primarily include case reports [40,41,42,43,44,45,46,47,49,50,51,52,53,54,55,56], with only one retrospective observational study [48]. The average patient age was 23.5 years (range 12–33 years), with a slight female predominance in case reports and more males in larger studies [40].

Most studies originated from Brazil, followed by Saudi Arabia [41,54] and Indonesia [47,53], with additional studies from Spain [40], Morocco [42], Thailand [48], India [49], Switzerland [50], Italy [52], and Germany [56].

The most common anomaly was Peg MLI, reported in thirteen studies [40,41,42,44,45,46,47,49,50,51,52,53,54,56], while microdontic lateral incisors (Mi MLIs) were noted in seven studies [40,41,43,44,46,48,55]. One study also described hypoplasia of central incisors and canines [40].

#### Treatment Approaches

Direct composite was applied in seven studies [40,41,45,46,49,53,55], while ceramic veneers were preferred in six studies [43,44,48,51,52,54].

Orthodontic treatment was used to align teeth, redistribute spaces, and prepare for prosthetic procedures [45,46,48,49,50,52,53,55], while bleaching was employed to enhance smile esthetics before restorations [43,45,46,51,54].

Crown lengthening was used in three studies to improve restoration with veneers or composites [43,48,51]. Tooth reshaping was employed as a temporary or definitive solution [42,43,48,51]. Injectable composite resin technique provided minimally invasive esthetics [41]. In two cases, MLIs were extracted, and orthodontics was used for space closure to improve esthetics and function. Prefabricated veneers were used in two studies [47,50].

### 3.6. Pre-Treatment Parameters

Table 5 indicates pre-treatment parameters for the studies of the second research stage.

Malocclusion details were not provided in several studies [40,41,43,50,51,53,54], while others reported diastemas among the anterior maxillary teeth [43,44,45,46,47,48].

Most patients had a Class I molar relationship [42,46,49,52,55], with skeletal classifications varying: Class I in three studies [46,52,55], Class II in one [56], and Class III in another [42]. Bimaxillary retrusion was noted in two cases [42,56]. Midline deviation appeared in various studies [42,49,56] and mandibular crowding was common [42,49,52,55].

Overjet was generally normal except for one case [56], and overbite was increased [49,52,55].

Regarding the diagnostic tests, photography was the most common diagnostic tool, with orthopantomography and cephalometric radiographs used in comprehensive evaluations [42,49,52,55,56]. Apical radiography was reported in some studies [48,50,55].

Diagnostic casts were widely used for occlusion visualization [41,43,44,45,47,48,49,50,51,53,54,55,56], while DSD appeared in four studies for esthetic planning [45,47,50,52].

Mock-up techniques were applied in five studies [41,43,45,51,52].

Bolton and dento-basal analyses were often not reported [40,41,43,44,51,53,54,55,56]. Benkaddour et al. [42] reported a 12 mm dento-basal discrepancy. Kalia et al. [49] identified a 3 mm space requirement for the maxillary arch and 5.5 mm for the mandibular arch, indicating mandibular tooth excess. DSD was used for esthetic planning in several studies [45,47,50,52].

### 3.7. Treatment Outcomes

Table 6 describes the primary and secondary outcomes of different treatment methods for studies included in the second research stage.

The esthetic assessment highlighted key factors such as color match, marginal discoloration, surface roughness, and esthetic integrity, with material choice significantly influencing outcomes. Some studies did not detail clinical evaluations [42,43,44,49], while others noted favorable esthetics [51,55,56]. Both ceramic veneer (CV) and composite restorations showed positive color match results [45,47,53], with “very good match” reported in some studies [40,54]. CV treatments provided natural color, excellent translucency, and youthful appearance [48,52]. Prefabricated veneers also demonstrated good color and shine [50]. Marginal discoloration was minimal or absent in most studies [41,48,52,53,54], with some reporting slight staining depending on composite resin types [40]. Good marginal adaptation was generally achieved [41,47,48,50,52], while surface roughness was consistently smooth [40,41,45,52,53,54]. Esthetic integrity was positively reported in most studies [40,41,45,46,47,48,52,53,54].

Most studies did not report detailed periodontal health findings [40,42,43,45,46,47,48,49,50,51,52,53,54,56]. Positive results with no soft tissue inflammation or BOP were noted in few studies [41] with a healthy periodontium observed in others [43,55].

No data are reported concerning TMJ signs and symptoms in the studies selected.

Studies combining conservative and orthodontic treatments showed improvements in dental alignment and occlusal relationships. Benkaddour et al. [42] reported corrected lower crowding and Class I canine/molar relationships. Kalia et al. [49] found improvements in crowding, deep bite, and incisor alignment. Perasso et al. documented lower crowding alignment and space redistribution for lateral incisors. Tanaka et al. [55] reported Class I relationships and good incisor inclination. Tausche et al. [56] achieved stable Class II occlusion with ideal overjet and overbite. Other studies did not provide specific occlusal data.

Follow-up periods varied, with shorter follow-ups at six months [48,53], ten months [44], one year [55] and one and a half years [49]. Mid-term follow-ups ranged from two years [40,41,45,49,52] to three years [50,56], while only one study offered a long-term follow-up of nine years [46]. Five studies did not provide follow-up data [42,43,47,51,54].

### 3.8. Quality Analysis

Regarding the qualitative analysis of the first search, the two RCT studies show a low risk [25,31], as described in Figure S1.

Among the non-randomized studies, eight articles are rated as moderate [1,23,24,28,32,33,34,35], five as serious [26,36,37,38,39] and three as critical [27,29,30], as described in Figure S2.

As for the quality assessment of the case reports included in the second search, a total of three articles scored 6 [48,49,52] and a total of thirteen articles scored 5 [41,42,43,44,45,46,47,50,51,53,54,55,56]. Table S3 summarizes the included case reports of research stage 2, detailing the responses to each question and the final score.

The non-randomized study of the second search is rated as serious, as described in Figure S3 [40].

The GRADE tool for formulating and grading recommendations demonstrates an evidence profile ranging from very low to low–moderate (Tables S4 and S5) [22].

## 4. Discussion

The congenital absence of the maxillary lateral incisor, particularly in its unilateral form, is common and often coexists with a contralateral Mi or Peg MLI [57]. This co-occurrence complicates treatment planning due to its impact on both esthetics and function, and despite its relative prevalence, the existing literature lacks high-quality studies providing clear guidance on standardized treatment protocols for its management, which typically involves evaluating whether to open or close the space for the UMLIA and determining whether to restore or replace the Mi or Peg MLI.

To address this gap and answer the PICO question, a dual search strategy was employed to embrace the full range of therapeutic options for managing UMLIA and the associated Mi or Peg MLI. This review included a range of heterogenous studies, each contributing different insights into pre-diagnostic parameters and clinical outcomes, with a focus on esthetics, periodontal health, function, and occlusion. By synthesizing findings from these diverse studies, a more comprehensive understanding of the available treatment approaches was sought, despite the variability in the study designs and outcome measures.

Regarding the treatment of UMLIA, our findings align with current clinical evidence. Other reviews have similarly observed that it is impossible to demonstrate the absolute superiority of either OSC or OSO techniques for UMLIA [9,58,59,60]. Instead, it is possible to observe that each approach presents distinct strengths in terms of esthetics, periodontal health, occlusion and TMJ disorders [61,62]. Consequently, each method may be better suited for different clinical situations and individual patients’ needs [63,64,65].

Esthetic outcomes varied across treatments. Several studies found that patients treated with OSO with IT or FDP typically experienced higher satisfaction regarding crown color and shape [1,27,28,32]. This is likely because prosthetic options allow for greater customization of appearance that better mimics the natural morphology of MLI, but still, this approach may result in subtle asymmetries in smile esthetics.

In contrast, other studies emphasized that OSC often achieves anterior symmetry and midline alignment, using the patient’s natural dentition to achieve a harmonious and natural smile [1]. Other authors [33,66] have noted that the natural color of the canine when moved into the position of MLI often matches well with adjacent teeth, resulting in a more authentic-looking smile compared to artificial crowns.

In terms of gingival color and soft tissue esthetics, OSC generally produces better results than OSO with IT [27,28]. Immediate implant restorations have demonstrated improvements in soft tissue esthetics [25], as, often, IT presents long-term biological complications, such as blue gingival discoloration and recession, with visible metal or porcelain abutments, particularly in patients with thin periodontal biotypes. Consequently, for patients that have a gummy smile, OSC may be the more desirable choice [27]. To address these esthetic challenges, some authors suggest shifting the issue to a less visible area, such as the posterior region, by creating space in the premolar zone [12,67].

From a periodontal perspective, this review’s data aligns with the existing literature, highlighting that OSC provides superior periodontal stability, particularly in plaque [1,24] and bleeding control [24,27,33]. Probing depth remained stable in both approaches, but OSC demonstrated fewer inflammatory complications and minimal gingival recession [24,32,33]. Conversely, OSO, particularly with implants, is more prone to gingival recession and challenges in maintaining interdental papilla [24,27,28]. These findings emphasize the periodontal advantages of OSC in promoting long-term stability and reducing complication compared to OSO [33,68,69]. In contrast, other authors comparing OSC and OSO + IT noted a higher incidence of gingival recession in correspondence with the first premolar in the OSC group and did not find significant differences between groups regarding papilla and gingival margin [70].

TMD issues were minimal, with no significant differences between treatment modalities. Functional outcomes were positive across treatments, with OSC showing reliable results and OSO achieving acceptable occlusal function [1,27,28]. These findings agree with the current literature [33,61]. The studies indicated that all treatment modalities provided effective occlusal function, with differences in the type of guidance achieved: functional in OSC-treated patients [24,33] and canine lateral guidance in OSO + IT [24].

The studies reviewed lack standardized pre-treatment parameters to guide the choice between OSC and OSO for UMLIA. Malocclusion classifications, imaging methods and Bolton analysis were inconsistently reported. Traditional indications for OSC, according to the literature, include cases with upper arch crowding, Class II malocclusion, significant lower arch crowding or protrusion of the lower incisors, and normally inclined teeth in a well-balanced profile along with canines that are favorable in shape, size, and color [9,12,71]. Conversely, OSO is typically recommended when there is pronounced maxillary spacing, no malocclusion with normal posterior intercuspation, Class III malocclusion with retrognathic profile or a marked size difference between cuspids and first premolars [72,73], with the purpose of increasing the anterior Bolton ratio, resulting in an improvement in the patient’s overjet [74,75].

Moreover, the choice of implants to replace UMLIA should be guided by an accurate assessment of the amount of space for the implant and the crown, considering the contralateral lateral incisor; the width of the alveolar ridge should be no less than 6 mm and the interradicular distance between adjacent teeth should leave a space of approximately 1.4 mm between the implant and the adjacent roots [14].

Additionally, some authors suggest that OSC can be successfully applied across all malocclusion types [76]. When chosen during adolescence, it offers the undeniable advantage of achieving definitive results by the end of orthodontic treatment, eliminating the need for additional interventions in adulthood, such as implants or prosthetic definitive solutions [72].

Concerning the management of Mi or Peg MLI, some studies recommend extraction due to its unfavorable size and shape and OSC with tooth reshaping of the canine [42,56]. In cases where extraction is not pursued, both ceramic and composite restoration showed positive outcomes [77]. Ceramic veneers are highly valued for their natural brilliance and translucency [43,44,48,51,54], while direct composites, like flowable composites and nanohybrids, are favored for their color matching and minimal invasiveness [40,41,45,49,53,55]; the use of the injection technique is well reported [41]. Some studies employed bleaching [43,45,51,52,54] and others used esthetic crown-lengthening procedures to improve gingival margin [43,48,51]. The use of wax-ups and/or mock-ups for treatment previsualization was largely found in facilitating communication between the practitioner and the patient in most of the studies, and some studies used DSD for esthetic planning and measurements. Both materials showed good marginal adaptation, esthetic integrity, and periodontal health with minimal inflammation. Occlusal improvements, with better alignment and stable relationships, were achieved; however, none of the included studies analyzed the possible repercussions or the initial status of the TMJ.

From the findings of this study, it is not possible to definitively establish the best therapeutic approach for treating UMLIA cases associated with Mi or Peg MLI. As some authors suggest [77], rather than proposing a single protocol, it is more effective to outline key considerations for treatment. These include clinical features related to facial, dental and tooth characteristics, as well as specific aspects of Mi or Peg MLI, such as cervical width, length, height, bucco-palatal position and occlusal relationships.

Our study shows a growing trend toward multidisciplinary treatment, combining minimally invasive dentistry and orthodontics to preserve periodontal health and esthetics. These approaches are enhanced by modern conservative techniques aimed at harmonizing the smile arch.

In this context, when a peg-shaped lateral incisor is present on one side and agenesis is present on the other, achieving esthetically symmetrical results can be challenging. Some authors advocate for combining OSC with a ceramic veneer [72] restoration on the peg-shaped incisor to enhance symmetry. Others suggest that the optimal approach may involve extracting the conoid incisor and performing bilateral OSC, enabling more consistent and symmetrical biomechanics. This strategy promotes proper midline alignment and effectively camouflages the anterior sector, leading to superior functional and esthetic outcomes [42,56].

The study has certain limitations, as follows:

- Although it includes numerous articles, the existing literature consists of heterogeneous studies with varying inclusion and sampling criteria.
- The first search incorporated studies of lower quality.
- Two separate searches were performed due to the presence of multiple non-standardized therapeutic approaches, which introduced complexity to the analysis.
- In the second search, case reports were included because of the lack of studies with greater scientific relevance.

It is recommended that future research focuses on prospective, randomized clinical trials with larger patient samples exhibiting both anomalies. Such studies should evaluate different treatments using control groups and assess long-term outcomes for esthetics, periodontal health, occlusion, and function.

## 5. Conclusions

The treatment of UMLIA combined with Mi or Peg MLI needs tailored solutions:

- A single standardized protocol cannot be universally applied; instead, the treatment approach (OSO or OSC) should be guided by the specific clinical situation and the individual needs of each patient.
- A multidisciplinary approach is essential for therapy planning.
- OSO with IT or FDP provides immediate esthetic results and patients report satisfaction.
- OSC with TR achieves excellent periodontal outcomes in the long term.
- OSO and OSC yield similar results in terms of TMJ signs and symptoms and favorable and stable occlusion.
- Both direct composite resin and indirect ceramic restorations provide comparable esthetic, periodontal, TMJ and occlusal outcomes for reshaping the MI or Peg MLI.
- Extracting the Mi or Peg MLI and performing bilateral OSC offers a more balanced approach to achieving greater symmetry in the smile arch.
- Due to the mentioned study limitations (heterogeneity of studies, low quality of included studies, inclusion of case reports), results should be interpreted with caution, emphasizing the need to promote new research with greater scientific evidence.

## Figures and Tables

**Table 1:** Study characteristics for studies included in research stage 1.

Author (Year) [Reference]	Country	Study Design	Sample M/F (Age)	Type of Agenesis	Treatment	Conclusions
AlRushaid et al., 2016 [23]	USA	Retrospective	G1:15: 6M/9F (16.5 ± 3.4 y) CG: 15: 6M/9F (16.08 ± 3.23 y)	UMLIA	OSO/OSC	OSC improves esthetics if width is insufficient
De Marchi et al., 2012 [24]	Brazil	Prospective	G1: 26: 6M/20F (24.95 y) G2: 20: 3M/17F (25.12 y) CG 22: 7M/15F (21.3 y)	UMLIA/BMLIA	G1:OSC + TR G2: OSO + IT	OSC better periodontal in thin biotype
Degidi et al., 2009 [25]	Italy	Randomized clinical trial	G1 and G2: 30 27M/33W (31.5 ± 11.8 y)	UMLIA	G1: immediate IT G2: one-stage IT	IT reliable, no significant bone loss
Garnett et al., 2006 [26]	UK	Retrospective	G1: 45: 14M/31F (17.6 y) CG:NR	UMLIA/BMLIA	OSO + RBBs	RBBs effective, staff experience matters
Hedmo et al., 2024 [27]	Sweden	Retrospective	G1 (early cohort): IT 22: 12M/10F (23.0 ± 28.0 y) OSC 22: 2M/20F (19.5 ± 31.5 y) G2 (latter cohort): IT 22: 8M/14F (24.6 ± 33.7 y) OSC 22: 8M/14F (20.5 ± 30.7 y) CG: NR	UMLIA/BMLIA	G1: OSO (all but three) + IT/OSC G2: IT/OSC	IT better for early cohort, OSC better crown length and BOP
Josefsson et al., 2019 [28]	Sweden	Retrospective	G1: 22: 8M/14F (24.6 to 33.7 y) G2: 22: NR (20.5 to 30.7 y) CG:NR	UMLIA/BMLIA	G1: OSO + IT: G2: OSC	OSC preferable
Kafantaris et al., 2020 [29]	Greece	Retrospective	G1: 8 G2: 31 G3: 3 11M/31F (M > 18 y; F > 17 y) CG: NR	UMLIA/BMLIA	G1: OSO +IT G2: OSO + RBBs G3: OSC + TR	Decision based on age and clinical characteristics; RBBs reliable
Lacarbonara et al., 2022 [30]	Italy	Prospective	G1: 35: 14M/21F (M:19.5 ± 2.2 W:18.5 ± 1.5 y) CG: NR	UMLIA/BMLIA	G1: OSO + IT	Good implant stability, no infraocclusion
Pithon et al., 2021 [31]	Brazil	Randomized controlled trial	G1: 22: 7M/15F (17 to 49 y) CG: 22: 6M/16F (17 to 49 y)	UMLIA/BMLIA	OSC + TR	OSC + TR: positive impact on OHRQoL
Robertsson et al., 2000 [1]	Sweden	Retrospective	G1: 30: 7M/23F (25.5 ± 7.5 y) G2: 20: 7M/13F (26.1 ± 6.2 y)	UMLIA/BMLIA	G1: OSC G2: OSO + RBBs	OSC stable and better accepted by patients
Roccuzzo et al., 2022 [32]	Denmark	Prospective	G1: 50: 17M/33F (21.2 ± 2.5 y) G2: 50: 24M/26F (21.8 ± 2.8 y)	UMLIA	G1 and G2: OSO + IT (Ø 2.9 mm and 3.3 mm)	Good esthetics and patient satisfaction
Rosa et al., 2016 [33]	Italy	Retrospective	G1: 26: 9M/17W (23 y 7 m) CG: 32: 12M/20W (17 y and 7 m)	UMLIA/BMLIA	OSC + TR	OSC maintains periodontal status
Sorrentino et al., 2023 [34]	Italy	Prospective	G1: 72: 37M/49W (19 to 46 y) CG: NR	UMLIA/BMLIA	OSO + IT	Effective implant–prosthetic treatment.
Sorrentino et al., 2024 [35]	Italy	Prospective	G1: 22: 7M/15W (18 to 37 y) CG: NR	UMLIA/BMLIA	OSO + IT	High success rate with digital workflow
Spinas et al., 2013 [36]	Italy	Prospective	G1: 30: 10M/20F (13–17 y) CG: NR	UMLIA/BMLIA	OSO + FRC-FPD	Good functional performance with FRC-FPD
Ulhaq et al., 2019 [37]	Scotland	Retrospective	G1: 15: 5M/10F (14.3 ± 1.4 y) G2: 29: 9M/20F (13.4 ± 2.6 y)	UMLIA/BMLIA	G1: OSC G2: OSO + RBBs	Adequate space influences OSO choice
Uribe et al., 2013 [38]	USA	Retrospective	G1: 11: 7M/4F (16.45 ± 5.76 y) CG: NR	UMLIA/BMLIA	OSO + IT	OSO may require bone grafting
Zarone et al., 2006 [39]	Italy	Prospective	G1 30:11M/19F (from 21 to 45 y) CG: NR	UMLIA/BMLIA	IT	Reliable and predictable IT outcomes

Abbreviations: G1, group 1; G2, group 2; G3, group 3; CG, control group; M, male; F, female; y, years; m, months; MLIA, maxillary lateral incisor agenesis; UMLIA, unilateral maxillary lateral incisor agenesis; BMLIA, bilateral maxillary lateral incisor agenesis; OSO, orthodontic space opening; RBBs, resin-bonded bridges; FRC-FPD, fiber-reinforced composite fixed partial dentures; IT, implant treatment; OSC, orthodontic space closure; TR, tooth re-contouring; TMD, temporomandibular disorder; NR, not reported; NS, non-significant; CBL, crestal bone level; OHRQoL, oral-health-related quality of life.

**Table 2:** Pre-treatment parameters for research stage 1.

Author (Year) [Reference]	Malocclusion	Diagnostic Tests	Bolton and Dento-Basal Analysis
AlRushaid et al., 2016 [23]	NR	NR	Reduced root and crown width of contralateral MLI in G1 vs. CG
De Marchi et al., 2012 [24]	NR	NR	NR
Degidi et al., 2009 [25]	NR	PAX, OPT, CBCT	NR
Garnett et al., 2006 [26]	Incisor Class I: 62.2%, Class II: 28.9%, Class III: 8.9%	NR	NR
Hedmo et al., 2024 [27]	NR	NR	NR
Josefsson et al., 2019 [28]	No Crowding	NR	Anterior space: G1: 50%/G2: 68%
Kafantaris et al., 2020 [29]	Molar Class I: 95%, Class II: 2.5%, Class III: 2.5%, Smile line high: 52%, low: 48%, crowding: 100%	PAX, OPT, Ceph	Maxillary spacing: 5%; canine–incisor size discrepancy: 19%
Lacarbonara et al., 2022 [30]	NR	PAX	NR
Pithon et al., 2021 [31]	Skeletal Class I, Angle Class I/II	NR	Space MD: G1: 6.35 mm/CG: 6.9 mm
Robertsson et al., 2000 [1]	NR	Ceph (39 patients)	Inferior space: G1: −2.1 mm/G2: +0.1 mm Maxillary space: G1: +2.7 mm/G2: +5.7 mm
Roccuzzo et al., 2022 [32]	NR	PAX	Space MD: G1: 5.9 to 6.3 mm/G2: 6.4 to 7.1 mm
Rosa et al., 2016 [33]	NR	PAX	NR
Sorrentino et al., 2023 [34]	NR	PAX, CBCT	NR
Sorrentino et al., 2024 [35]	NR	PAX, CBCT	NR
Spinas et al., 2013 [36]	NR	NR	NR
Ulhaq et al., 2019 [37]	Overjet ≥ 2 mm G1: 73%/G2: 79%, Molar Class I: G1:40%/G2:62%, Class II: G1:60%/G2:31%, Class III: G1: 0/G2:7%	NR	Adequate space MD: G1: 13%/G2: 55%; Inadequate: G1: 87%/G2: 45%
Uribe et al., 2013 [38]	NR	CBCT	NR
Zarone et al., 2006 [39]	NR	PAX, CBCT	NR

Abbreviations: G1, group 1; G2, group 2; G3, group 3; CG, control group; NR, not reported; MLI, maxillary lateral incisor; PAX, periapical X-ray; OPG, orthopantomography; Ceph, cephalometric X-ray; CBCT, cone-beam computed tomography system; CR, class relationship; MD, mesio-distal distance between the canine and the central incisor.

**Table 3:** Treatment parameters for studies included in research stage 1.

Author (Year) [Reference]	Esthetic Assessment	Periodontal Assessment	TMD Signs and Symptoms Assessment	Occlusion Assessment	Follow-Up
AlRushaid et al., 2016 [23]	NR	LP ARW of contralateral MLI 1 mm narrower in G1 vs. CG	NR	NR	NR
De Marchi et al., 2012 [24]	NR	PI: G1: 61%, G2: 52%; PD: 3 mm BOP: G1: 18%, G2: 7% GR: absent in thick biotype PaI: G2 more changes in mesial papilla	No TMD: G1: 85%, G2: 75%, CG: 91% Low myofascial pain and disk displacement	Protrusive guidance and canine lateral guidance: G1: 57%, G2: 80%, group disocclusion G1: 43%, G2: 20%	G1: 3.90 ± 3.48 y G2: 3.54 ± 2.39 y
Degidi et al., 2009 [25]	Better soft tissue esthetics in G1	PD, BOP and bone loss: NS	NR	NR	3 y
Garnett et al., 2006 [26]	NR	NR	NR	Harmonious excursive movements, intercuspidal contact	>100 m
Hedmo et al., 2024 [27]	Non-acceptable crown color: IT 0%, OSC G1: 20.5%, G2: 0%; Abnormal crown length higher in IT. Better gingival color with OSC. Midline deviation maxilla: IT: G1: 45.5%, G2: 22.5%, OSC: G1: 41%, G2: 36.5%	BOP: IT: G1: 85.5%, G2: 53%, OSC: G1: 35.5%, G2: 5.5%; Suppuration: IT: G1: 0%, G2: 15%, OSC: 0%; GR: IT: G1: 28.5%, G2: 11.5%, OSC: G: 14.5%, G2 11%; Papilla defect: IT: G1: 68%, G2: 38%, OSC: G1: 38%, G2: 22%	NR	Angle Class I: IT: G1: 77%, G2: 82%, OSC: G1: 86%, G2: 37%; Overjet: IT: G1: 3 mm, G2: 3.1 mm, OSC: G1: 2.8 mm, G2: 2.5 mm; Overbite: IT: G1: 3.3 mm, G2: 3 mm, OSC: G1: 3.5 mm, G2: 2.4 mm; Proclination of incisors: IT: G1: 45.5%/G2: 0%, OSC: G1: 13.5%, G2: 18%	5 y
Josefsson et al., 2019 [28]	Non-acceptable crown color: G1: 0%, G2: 21%. Abnormal crown length: G1: 61%, G2: 15%. Gingival color issues: G1: 61%, G2: 9%. Strained lip closure: G1: 23%, G2: 4%. Midline deviation maxilla: G1: 45%, G2: 41%. Non-acceptable appearance when smiling: G1: 32%, G2: 4%.	BOP: G1 25%, G2 35%; GR: G1 29%, G2 15%; Papilla defect: G1 71%, G2 59%	NR	Angle Class I: G1: 77%, G2: 86%; Overjet: G1: 3 mm, G2: 2.8 mm; Overbite: G1: −1.5 and 7 mm, G2: 0 and 7 mm; Proclination of incisors: G1: 32%, G2: 4%	5 y
Kafantaris et al., 2020 [29]	G1: 12.5% unsatisfactory gingival zenith after 4 y. G2: 58% good esthetic outcomes.	G1: 12.5% peri-implantitis, (stage IV grade C) after 10 y	No significant differences	NR	G1, G2: yearly G3: no follow up
Lacarbonara et al., 2022 [30]	Satisfactory results	BOP and bone resorption: NS No vertical bone changes	NR	No infraocclusion	10 y
Pithon et al., 2021 [31]	NR	NR	NR	NR	1 y
Robertsson et al., 2000 [1]	Satisfaction crown color/shape: G1: 45%, G2: 81%. Space satisfaction: G1: 80%, G2: 75%. Good symmetry: G1: 67%, G2: 50%.	PI and BOP in anterior vs. premolar region	No significant TMJ issues; No pain, sounds, or muscle tenderness	Angle Class I: G1: 65%, G2: 67%; Overjet: G1: 2.3 mm, G2: 2.1 mm; Overbite: G1: 3.0 mm, G2: 2.5 mm ANB: G1: 3.3°, G2 2.1°; SNA: G1: 81.4°, G2: 79.2°; SNB: G1: 78.1°, G2: 77.1°	G1: 7.1 ± 3.3 y G2: 7.2 ± 3.8 y
Roccuzzo et al., 2022 [32]	Excellent crown color: G1: 47.8%, G2: 26.2%. Excellent crown shape: G1: 69.9%, G2: 97.6%. Soft tissue color: G1: 39.1%, G2: 35.7%. Symmetry satisfaction: G1: 41.3%, G2: 61.9%.	PI: G1: 15%, G2: 12%; PD: G1: 2.55 ± 0.41 mm, G2: 2.50 ± 0.45 mm; Suppuration: G1: 2%, G2: 2%; Mesial PaI excellent: G1: 65.2%, G2: 73.2%; Distal PaI excellent: G1: 93.3%, G2: 100%;	NR	NR	1 y
Rosa et al., 2016 [33]	NR	PD < 4 mm, few bleeding sites, GR not significant	Tooth grinding: G1 > CG	OSC: group function CG: canine-raised occlusion	10 y
Sorrentino et al., 2023 [34]	No significant difference between AGC crowns and all-ceramic crowns	Healthy and stable peri-implant soft tissue	NR	Functional outcomes: NS	NR
Sorrentino et al., 2024 [35]	Fully satisfactory for the patients	Healthy peri-implant soft tissue, good osteointegration and bone stability	NR	Fully satisfactory functional outcomes	1 y, 2 y
Spinas et al., 2013 [36]	Acceptable esthetic outcomes	NR	NR	Acceptable function	5 y with annual check-up
Ulhaq et al., 2019 [37]	NR	NR	NR	NR	NR
Uribe et al., 2013 [38]	NR	ABW by 17% to 25%; increased labial concavity; minimal soft tissue changes	NR	NR	NR
Zarone et al., 2006 [39]	NR	Optimal PI and BOP, bone resorption satisfactory values, Pai improved from satisfactory to optimal	NR	Reliable and predictable functional outcomes	G1: 2–3 y CG: yearly

Abbreviations: TMD, temporomandibular disorder; ARW, alveolar ridge width; G1, group 1; G2, group 2; G3, group 3; CG, control group; y, years; m, months; TMJ, temporomandibular joint; NR, not reported; NS, non-significant; PI, plaque index; PD, probing depth; BOP, bleeding on probing; GR, gingival recession; PaI, papilla index; AGC, Auro-Galvan Crown.

**Table 4:** Study characteristics for studies included in research stage 2.

Author (Year) [Reference]	Country	Study Design	Sample M/F (Age)	Type of Anomaly of MLI	Treatment	Conclusions
Alonso et al., 2012 [40]	Spain	Retrospective	G1: 21: 14M/7F (22.5 ± 8.2 y) CG: NR	Peg/Mi	DCFC	Minimally invasive, ideal for growing patients
Alyahya et al., 2024 [41]	Saudi Arabia	Case report	1: F (24 y)	Peg/Mi	DCFC with ICRT	Effective esthetics and functional results
Benkaddour et al., 2017 [42]	Morocco	Case report	1: M (17 y)	Peg	Ex MLI + OSC	Balanced function and esthetics, requires careful planning
da Cunha et al., 2017 [43]	Brazil	Case report	1: F (20 y)	Mi	CL + B + temporary TR + CV	Conservative approach for smile enhancement
da Cunha et al., 2018 [44]	Brazil	Case report	1: M	Peg	Elastics separators + CV	Efficient space redistribution, maintains periodontal health
de Oliveira et al., 2022 [45]	Brazil	Case report	1: M (30 y)	Peg	B + OT + DCFC	Minimally invasive, cost-effective
Francisconi et al., 2012 [46]	Brazil	Case report	1: F (18 y)	Peg/Mi	OT + TR + B+ TR	Multidisciplinary approach ensures long-term satisfaction
Irmaleny et al., 2024 [47]	Indonesia	Case report	1: F (32 y)	Peg	Prefabricated Veneers	Quick, customizable, cost-effective
Ittipuriphat et al., 2013 [48]	Thailand	Case report	1: F (21 y)	Mi	CL + OT + TR + CV + retainer at night	Multidisciplinary approach for minimal veneer prep
Kalia et al., 2015 [49]	India	Case report	1: F (22 y)	Peg	OT + DCFC	Multidisciplinary approach for minimal veneer prep
Parisini et al., 2017 [50]	Switzerland	Case report	1: F (16 y)	Peg	OT + Prefabricated Veneers	Cost-effective for patients on a budget
Pena et al., 2009 [51]	Brazil	Case report	1: F (22 y)	Peg	CL + B + temporary TR + CV	Interdisciplinary approach for excellent results
Perasso et al., 2018 [52]	Italy	Case report	1: F (27 y)	Peg	OT + B + CV + retention	Shared diagnosis enhances treatment planning
Putri et al., 2022 [53]	Indonesia	Case report	1: M (24 y)	Peg	OT + CL+ DCFC	Preserved space and esthetic outcomes
Refeai et al., 2023 [54]	Saudi Arabia	Case report	1: M (33 y)	Peg	B + CAD-CAM CV	Reliable esthetic solution, tailored to patient needs
Tanaka et al., 2020 [55]	Brazil	Case report	1: M (19.5 y)	Mi	OT + DCFC	Multidisciplinary approach for esthetics and occlusion
Tausche et al., 2008 [56]	Germany	Case report	1: F (12 y)	UMLIA/Peg	OSC + Ex of Pegs	Favorable esthetics and long-term occlusal stability

Abbreviations: G1, group 1; M, male; F, female; y, years; m, months; MLI, maxillary lateral incisor; B, bleaching; CV, ceramic veneer; DCFC, direct composite full coverage; ICRT, injectable composite resin technique; CL, esthetic crown lengthening; Ex, extraction; OT, orthodontic treatment; OSC, orthodontic space closure; TR, tooth re-contouring; UN, unclear; R, right; L, left; Peg, maxillary peg-lateral incisor; Mi, microdontic.

**Table 5:** Pre-treatment parameters for studies included in research stage 2.

Author (Year) [Reference]	Malocclusion	Diagnostic Tests	Bolton and Dento-Basal Analysis
Alonso et al., 2012 [40]	NR	Ph, no RX	NR
Alyahya et al., 2024 [41]	NR	Ph, Videos, Casts, Wax-up, Mock-up	NR
Benkaddour et al., 2017 [42]	Class I molar, II canine, Skeletal Class III	Ph, OPG, Ceph	12 mm dento-basal discrepancy
da Cunha et al., 2017 [43]	NR	Ph, Casts, Wax-up, Mock-up	NR
da Cunha et al., 2018 [44]	Multiple diastemas	Ph, Casts, Wax-up	NR
de Oliveira et al., 2022 [45]	Multiple diastemas, crowding	Ph, Casts, DSD, Mock-up	Yes, with DSD
Francisconi et al., 2012 [46]	Class I dental, Multiple diastemas	Ph	Yes
Irmaleny et al., 2024 [53]	Multiple diastemas	Ph, Casts, DSD	Yes, with DSD
Ittipuriphat et al., 2013 [48]	Multiple diastemas	Ph, Apical, Casts, Wax-up	Yes
Kalia et al., 2015 [49]	Class I molar and canine, crowding, overjet 1 mm, overbite 5.5 mm; deviation lower midline, exaggerated curve of Spee	Ph, OPG, Ceph, Casts	Maxillary arch: −3 mm Mandibular arch: −5.5 mm Bolton analysis: mandibular tooth excess
Parisini et al., 2017 [50]	NR	Ph, OPG, Apical, Casts, DSD	Yes, with DSD
Pena et al., 2009 [51]	NR	Ph, Casts, Wax-up, Mock-up	NR
Perasso et al., 2018 [52]	Class I dental/skeletal, deep bite, short face	Ph, OPT, Ceph, DSD, Wax-up, Mock-up	Yes, with DSD
Putri et al., 2022 [53]	NR	Ph, Casts, Wax-up	NR
Refeai et al., 2023 [54]	NR	Ph, Casts	NR
Tanaka et al., 2020 [55]	Class I skeletal/dental, normal overjet, overbite 6 mm, mild misalignment	Ph, OPG, Apical, Ceph, Casts	NR
Tausche et al., 2008 [56]	Class II molar and skeletal, retrognathic maxilla and mandible, overjet 7 mm, overbite 4 mm	Ph, OPG, Ceph, Casts	NR

Abbreviations: NR, not reported; Ph, photography; Rx, radiography; DSD, digital smile design analysis; PAX, periapical X-ray; OPG, orthopantomography; Ceph, cephalometric X-ray.

**Table 6:** Treatment outcomes for studies included in research stage 2.

Author (Year) [Reference]	Esthetic Assessment	Periodontal Assessment	TMD Signs and Symptoms Assessment	Occlusion Assessment	Follow-Up
Alonso et al., 2012 [40]	95% very good color match, 9.5% obvious marginal discoloration, 57% very good marginal adaptation, 57% smooth surface, good integrity	NR	NR	NR	2 y, 10 y, 11 y
Alyahya et al., 2024 [41]	no marginal discoloration, good marginal adaptation, smooth surface, good integrity	No inflammation, healthy soft tissue	NR	NR	2 y
Benkaddour et al., 2017 [42]	NR	NR	NR	Corrected crowding, Class I canine/molar, alignment of midlines; good lateral occlusion	NR
da Cunha et al., 2017 [43]	NR	NR	NR	NR	NR
da Cunha et al., 2018 [44]	NR	Healthy periodontium	NR	NR	10 m
de Oliveira et al., 2022 [45]	good color match, smooth surface, good integrity	NR	NR	Overbite still present	2 y
Francisconi et al., 2012 [46]	color mismatch and marginal discoloration at 9 y recall visit, good integrity	NR	NR	NR	9 y
Irmaleny et al., 2024 [47]	good color match and marginal adaptation, smooth surface, good integrity	NR	NR	NR	NR
Ittipuriphat et al., 2013 [48]	natural color match with excellent incisal translucency, youthful characterization, no marginal discoloration, good marginal adaptation and integrity	NR	NR	NR	6 m
Kalia et al., 2015 [49]	NR	NR	NR	Corrected crowding, deep bite, midlines and curve of Spee, ideal overjet and overbite	1 y and 6 m
Parisini et al., 2017 [50]	natural color match with high translucency, good marginal adaptation	NR	NR	NR	3 y
Pena et al., 2009 [51]	NR	NR	NR	NR	NR
Perasso et al., 2018 [52]	natural and brilliant color match, no marginal discoloration, good marginal adaptation, smooth surface, good integrity	NR	NR	Corrected incisors inclination, crowding, reduced overbite, better lip support	2 y
Putri et al., 2022 [53]	good color match and shine smooth surface, marginal discoloration and good integrity	NR	NR	NR	Every 6 m
Refeai et al., 2023 [54]	very good color match: very good, no marginal discoloration, smooth surface, good integrity	NR	NR	NR	NR
Tanaka et al., 2020 [55]	NR	Healthy gingival and periodontal status	NR	Class I relationship, well-maintained profile	1 y
Tausche et al., 2008 [56]	NR	NR	NR	Stable Class II occlusion, ideal overjet and overbite	3 y

Abbreviations: NR, not reported; y, years; m, months.

## Data Availability

The data presented in this study are available in the article.

## Supplementary Materials

The following supporting information can be downloaded at https://www.mdpi.com/article/10.3390/dj13040169/s1: Table S1: Search strategy for each database for the first search; Table S2: Search strategy for each database for the second search; Figure S1: Risk of bias of the included RCT study designs according to Rob 2, the Cochrane Collaboration’s risk-of-bias assessment tool; Figure S2: Risk of bias of the included prospective and retrospective study designs of the first search according to ACROBAT-NRSI, Cochrane Risk of Bias Assessment tool for Non-randomized Studies of Interventions; Table S3: Case reports included for the second search (critical appraisal); Figure S3: Risk of bias of the included retrospective study designs of the second search according to ACROBAT-NRSI, Cochrane Risk of Bias Assessment tool for Non-randomized Studies of Interventions; Table S4: Grading of Recommendations, Assessment, Development, and Evaluation (GRADE) analysis for the studies included in the first search; Table S5: Grading of Recommendations, Assessment, Development, and Evaluation (GRADE) analysis for the studies included in the second search.
