# Supplementary material for: Treatment Options for Unilateral Agenesis of the Maxillary Lateral Incisor Combined with Contralateral Microdontic or Peg-Shaped Lateral Incisor: A Systematic Review
Source: Dent J (Basel). 2025 Apr 17;13(4):169. doi: 10.3390/dj13040169 (PMC12026457; doi:10.3390/dj13040169)
Supplement: Supplementary file 1 [file dentistry-13-00169-s001.zip › dentistry-3545909-supplementary.pdf]

Treatment Options for Unilateral Agenesis of the Maxillary Lateral incisor combined with Contralateral Microdontic or Peg-shaped Lateral incisor: A Systematic Review

Dentistry Journal

Federica Bitonto 1, Alessio Verdecchia 1,*, Massimiliano Lombardo 1, Erica Lipani 1, Claudia Dettori 2 and Enrico Spinas 1,*

[1] Department of Surgical Sciences, Postgraduate School in Orthodontics, University of Cagliari, 09124 Cagliari, Italy; federibit@gmail.com (F.B.); info@massimilianolombardo.com (M.L.); erica.lipani@outlook.it (E.L.);

[2] Department of Surgical Sciences, School of Dental Medicine, University of Cagliari, 09124 Cagliari, Italy;

claudia.dettori@gmail.com (C.D.)

* Correspondence: verdecchia.belli.a@gmail.com(A.V); enricospinas@tiscali.it (E.S.)

Supplementary Information

Table S1. Search strategy for each database for the first research.

Database	Search Strategy	Number of results
Scopus	(TITLE-ABS-KEY (lateral AND incisor AND agenesis) OR TITLE-ABS-KEY (unilateral AND agenesis)) AND TITLE-ABS-KEY (maxillary AND lateral AND incisor) AND TITLE-ABS-KEY (treatment)	195
Web of Science	(ALL=(lateral incisor agenesis) OR ALL=(unilateral agenesis)) AND ALL=(maxillary lateral incisor) AND ALL=(treatment)	185
Embase	('lateral incisor agenesis' OR 'unilateral agenesis') AND 'maxillary lateral incisor' AND 'treatment'	38
Pubmed	((lateral incisor agenesis) OR (unilateral agenesis)) AND (maxillary lateral incisor) AND (treatment)	687
Cochrane Central Register of Controllled Trials	('lateral incisor agenesis' OR 'unilateral agenesis') AND 'maxillary lateral incisor' AND 'treatment'	3

Table S2. Search strategy for each database for the second research.

Database	Search Strategy	Number of results
Scopus	(TITLE-ABS-KEY (peg-shaped AND lateral AND incisor) OR TITLE-ABS-KEY (conoid AND incisor) OR TITLE-ABS-KEY (conoid AND teeth) OR TITLE-ABS-KEY (microdontia AND lateral AND incisor)) AND TITLE-ABS-KEY (treatment)	119
Web of Science	(ALL=(peg-shaped lateral incisor) OR ALL=(conoid incisor) OR ALL=(conoid teeth) OR ALL=(microdontia lateral incisor)) AND ALL=(treatment)	86
Embase	('peg-shaped lateral incisor' OR 'conoid incisor' OR 'conoid teeth' OR 'microdontia lateral incisor') AND 'treatment'	12
Pubmed	((peg-shaped lateral incisor) OR (conoid incisor) OR (conoid teeth) OR (microdontia lateral incisor)) AND (treatment)	119

Cochrane Central Register of Controlled Trials	((peg-shaped lateral incisor) OR (conoid incisor) OR (conoid teeth) OR (microdontia lateral incisor)) AND (treatment)	2

Figure S1. Risk of bias of the included RCT studies design according to the RoB 2, Cochrane Collaboration's risk of bias assessment tool.

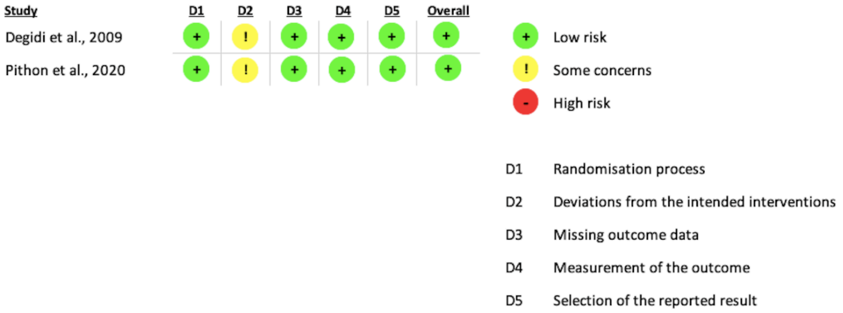

Figure S2. Risk of bias of the included prospective and retrospective studies design of the first research according to ACROBAT-NRSI, Cochrane Risk of Bias Assessment tool for Non-randomized Studies of Interventions.

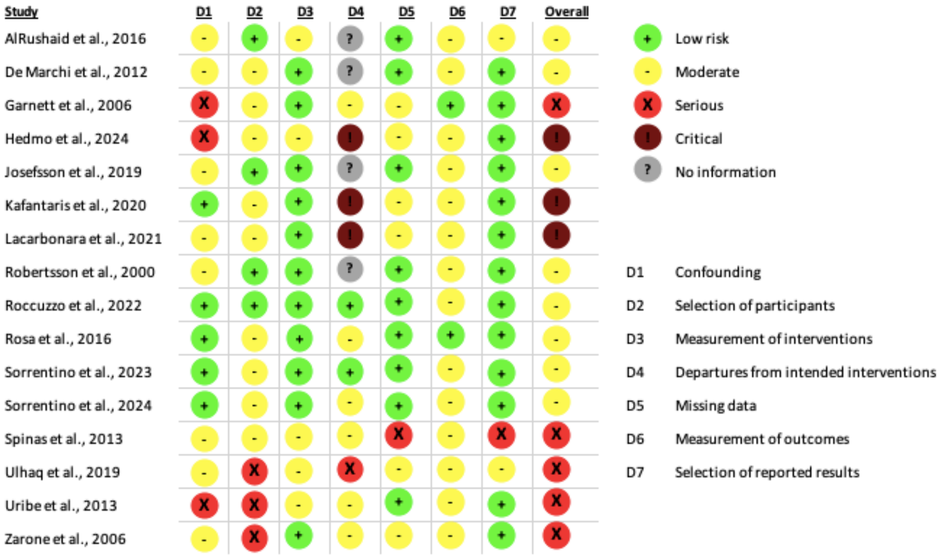

Table S3. Case reports included for the second research critical appraisal.

**Author (year) [Reference]**	**DC**	**PH**	**CCC**	**DT**	**TP**	**PICC**	**AEI**	**TL**	**Score (Y)**

Al Refeai et al., 2023 [15]	Yes	Unclear	Yes	Unclear	Yes	Yes	Yes	Yes	5
Alyahya et al., 2024 [16]	Unclear	Yes	Yes	Unclear	Yes	Yes	No	Yes	5
Benkaddour et al., 2017 [17]	Unclear	No	Yes	Yes	Yes	Yes	Unclear	Yes	5
da Cunha et al., 2017 [18]	Unclear	Yes	Yes	Unclear	Yes	Yes	No	Yes	5
da Cunha et al., 2018 [19]	Unclear	No	Yes	Unclear	Yes	Yes	Yes	Yes	5
de Oliveira et al., 2022 [20]	Unclear	No	Yes	Unclear	Yes	Yes	Yes	Yes	5
Francisconi et al., 2012 [21]	Unclear	No	Yes	Unclear	Yes	Yes	Yes	Yes	5
Irmaleny et al., 2024 [22]	Unclear	Yes	Yes	Unclear	Yes	Yes	No	Yes	5
Ittipuriphat et al., 2012 [23]	Unclear	Yes	Yes	Yes	Yes	Yes	No	Yes	6
Kalia et al., 2015 [24]	Unclear	Yes	Yes	Yes	Unclear	Yes	Yes	Yes	6
Parisini et al., 2017 [25]	Unclear	No	Yes	Yes	Yes	Yes	Unclear	Yes	5
Pena et al., 2009 [26]	Unclear	Yes	Yes	No	Yes	Yes	Unclear	Yes	5
Perasso et al., 2018 [27]	Unclear	No	Yes	Yes	Yes	Yes	Yes	Yes	6
Putri et al., 2022 [22]	Unclear	Yes	Yes	Unclear	Yes	Yes	Unclear	Yes	5
Tanaka et al., 2020 [28]	Unclear	Yes	Yes	Yes	Unclear	Yes	Unclear	Yes	5
Tausche et al., 2008 [29]	Unclear	No	Yes	Yes	Yes	Yes	Unclear	Yes	5

Abbreviations: DC, demographic characteristics; PH, patient history; CCC, current clinical condition; DT, diagnostic tests; TP, treatment procedure; PICC, post-intervention clinical condition; AEI, adverse events identified; TL, take away lesson.

Figure S3. Risk of bias of the included retrospective studies design of the second research according to ACROBAT-NRSI, Cochrane Risk of Bias Assessment tool for Non-randomized Studies of Interventions.

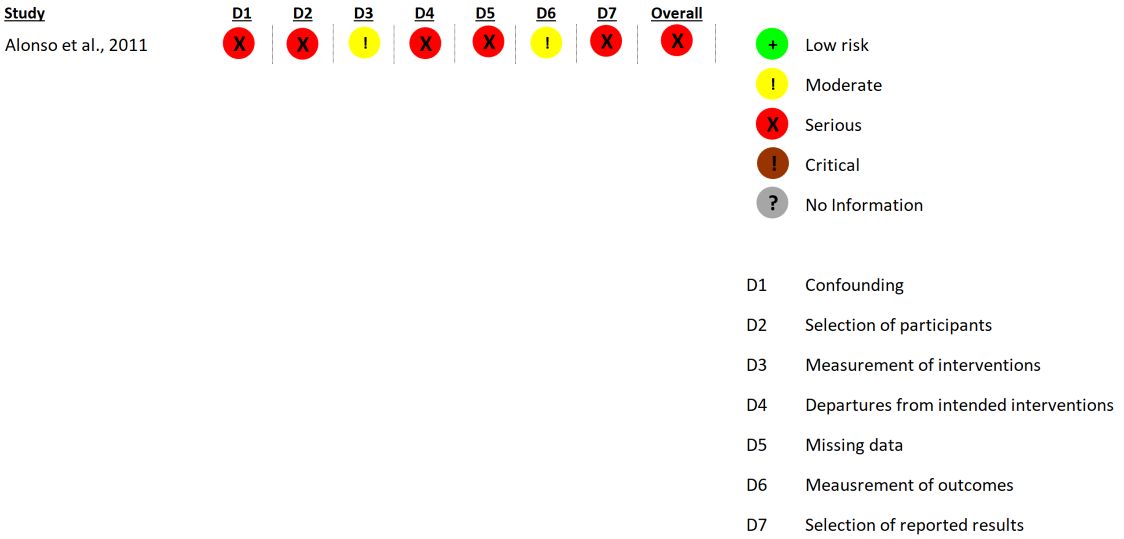

Table S4. Grading of Recommendation, Assessment, Development, and Evaluation (GRADE) analysis for the studies included in the first research.

**Certainty of assessment**							**Certaint y**
Nº of studies	Stud y	Risk of bias	Inconsisten cy	Indirectnes s	Imprecisio n	Other consideratio	
**IT (Implant Treatment)**							
**2** [24, 26, 38]	RCT, P	Serious	Not Serious	Not serious	Serious	None	Low ⨁◯◯◯
**OSO + IT (Orthodontic Space Opening + Implant Treatment)**							
**8** [23, 27, 28, 29,31,33, 34, 37]	6 P, 2R	Serious	Not Serious	Not serious	Serious	None	Low
**OSO + RBBs (Orthodontic Space Opening + Resin Bonded Bridges)**							
**4** [25, 28,1, 36]	4R	Serious	Not Serious	Not serious	Serious	None	Very Low ⨁◯◯◯
**OSO + FRC- FPD (Orthodontic Space Opening + Fiber reinforced composite-Fixed partial**							
**1** [35]	P	Serious	Not Serious	Not serious	Serious	None	Very Low ⨁◯◯◯
**OSC (Orthodontic Space Closure)**							
**5** [23,30,1,32,36]	1P, 4 R	Modera te	Not Serious	Not serious	Serious	None	Low -Moderat e

**OSC / OSO (Orthodontic Space Closure or Opening)**							
**2** [22, 26]	2R	Serious	Not Serious	Not serious	Serious	None	Very Low ⨁◯◯◯

Abbreviations: RCT: randomized clinical trial; P: prospective study, R: retrospective study.

Table S5. Grading of Recommendation, Assessment, Development, and Evaluation (GRADE) analysis for the study included in the second research.

**Certainty of assessment**							**Certaint y**
Nº of studies	Stud y [illegible]	Risk of bias	Inconsisten cy	Indirectnes s	Imprecisio n	Other consideratio	
**1** [39]	R	Serious	Not Serious	Not serious	Serious	None	Low ⨁◯◯◯
